# Turkish Adaptation of the Psychometric Properties of the ‘Nursing Learning Self‐Efficacy Scale’ for Nursing Students

**DOI:** 10.1111/ijn.70012

**Published:** 2025-03-25

**Authors:** Özlem Şahin Akboğa

**Affiliations:** ^1^ Nursing Department, Faculty of Health Sciences University of Yozgat Bozok Yozgat Turkey

**Keywords:** nursing students, psychometric testing, self‐efficacy, validity and reliability

## Abstract

**Background:**

Self‐efficacy is an important factor in nursing students' academic success. Thus, task‐dependent self‐efficacy should be evaluated in terms of learning. Valid and reliable tools are needed to measure learning self‐efficacy in different societies.

**Aim:**

The study aimed to test the ‘Nursing Learning Self‐Efficacy Scale’ psychometric properties in the Turkish population.

**Methods:**

It is a methodological study of instrument translation and validation. The psychometric properties of the Nursing Learning Self‐Efficacy Scale were tested with 345 nursing students. For reliability, 50 nursing students were included in the retest. The study was conducted between May and June 2024 in a Health Sciences Faculty located in the Central Anatolia region of Türkiye.

**Findings:**

The Content Validity Index of the scale was determined as 0.95 for linguistic compatibility and 0.92 for content compatibility. Exploratory factor analysis revealed a four‐dimensional factor structure with a cumulative variance contribution rate of 62.84%, and 20 items in the scale were included in the analysis. In the confirmatory factor analysis of the scale, the fit indices for the classes were acceptable. The Cronbach's alpha coefficient for Turkish culture was determined to be 0.926.

**Conclusion:**

The Turkish version of the Nursing Learning Self‐Efficacy Scale is a valid and reliable assessment tool for measuring self‐efficacy in the learning skills of nursing students.

**Summary**

**What is already known about this topic?**

- Nursing students need self‐efficacy to be successful in learning the nursing profession.
- There is a big gap in the literature where self‐efficacy is evaluated in many areas, but self‐efficacy in nursing education cannot be measured.
- In previous studies, self‐efficacy levels of individuals in different fields have been examined. This study revealed the importance of evaluating self‐efficacy in learning the nursing profession.

**What this paper adds?**

- The study provides an opportunity to evaluate Turkish nursing students' self‐efficacy in learning the profession.
- This study provides comprehensive, applicable and acceptable information for assessing Turkish nursing students' self‐efficacy in learning the profession.

**The implications of this paper:**

- This study provides a standardized tool for assessing nursing students' self‐efficacy in learning the nursing profession. This study can serve as a basis for further studies with a larger sample in Türkiye and other countries.

## 1 Introduction

Nursing competence is the ability of nurses to fulfil their roles (Fukada 2018). Nursing has many different areas of competence, including knowledge, skills, values, attitudes, communication, collaboration, critical thinking and innovation (Liu et al. 2021). Nursing students' sense of self‐efficacy should be established to gain practical clinical skills during education and fulfil their roles independently and competently in nursing (Shorey and Lopez 2021). Academic achievement, as well as health, organizational, interpersonal and emotional functioning, has been associated with self‐efficacy (Gerbino 2020). Bandura (1977) stated that self‐efficacy is an expected outcome that can be used to evaluate the effects of learning. Self‐efficacy is a factor that contributes significantly to student performance and has the strongest effect among the 50 factors that affect students' academic achievement (Richardson et al. 2012). Positive self‐efficacy increases students' motivation and willingness to accomplish new and challenging tasks. In contrast, negative self‐efficacy causes individuals to be unable to act on their own initiative or to leave a task unfinished (Karaoglan Yilmaz 2022). Nursing colleagues' attitudes and clinical educators' skills in clinical learning environments can positively affect nursing students' self‐efficacy (Shehadeh et al. 2020). The factors significantly associated with the clinical competencies of undergraduate nursing students are professional interest, self‐efficacy and clinical learning environments, and these factors account for 36.1% of the total variance explained. Self‐efficacy has been reported to play a mediating role between clinical learning environments and clinical competence (Yu et al. 2021). This situation revealed that self‐efficacy may positively affect the formation of professional identity among nursing students. Meanwhile, self‐efficacy was reported to mediate the relationship between professional identity and competence, with self‐efficacy accounting for 52% of the total effect on professional identity formation (Yao et al. 2021). Farokhzadian et al. (2020) also determined a significant relationship between the health‐promoting behaviours of nursing students and self‐efficacy and academic success. Nursing students need to transfer knowledge to practice accurately and effectively (von Colln‐Appling and Giuliano 2017). Self‐efficacy is an essential source of motivation for the development of nursing students (Hassankhani et al. 2015) and is critical for their performance in their roles (Mohmmdirezi et al. 2015). The self‐efficacy of nursing students is related to many learning issues.

Bandura (1997) stated that self‐efficacy is situation specific. This situation means a person with high self‐efficacy in one area has less self‐efficacy in others. This situation has led to the development of self‐efficacy assessment tools in different areas (general self‐efficacy [Gozum and Aksayan 1999; Schwarzer 1999]; self‐efficacy in resuscitation [Roh and Issenberg 2014]; and self‐efficacy for clinical assessment [Zengin et al. 2014]) aiming to measure the self‐efficacy of the individual. These scales evaluate an individual's field‐specific self‐efficacy. Students' perceptions of their learning experiences can significantly affect their learning self‐efficacy (Tusianah et al. 2021). Learning self‐efficacy was investigated as a predictor of the clinical skills performance of nursing students (Alosaimi 2021). Learning self‐efficacy (Bandura 1991), which is important for students' ability to achieve learning goals, has been recognized as a factor that affects students' learning performance (Alosaimi 2021). Therefore, nursing students' self‐efficacy in learning the profession must be evaluated separately. Yiin et al. (2024) developed the ‘Nursing Learning Self‐Efficacy (NLSE) Scale’ to examine the factors contributing to nursing students' self‐efficacy in learning and to promote a more comprehensive understanding of their needs and areas for development in both cognitive and practical aspects of their education. This scale has not yet been tested in different cultures. Therefore, the scale needs to be retested and validated in other cultures.

In line with this information, this study aimed to verify the psychometric properties of the Turkish version of the NLSE Scale, which measures nursing students' self‐efficacy in learning the nursing profession. This research will provide a valid and reliable measurement tool to determine nursing students' learning self‐efficacy and will significantly contribute to other nursing studies.

### 1.1 Research Hypotheses

Hypothesis 1 *The Nursing Learning Self‐Efficacy Scale is a valid and reliable for the Turkish population*.

## 2 Materials and Methods

### 2.1 Research Design and Sample

This study was conducted using a methodological design between May and June 2024 with nursing students studying at a university in the Central Anatolia region of Türkiye. The study population consisted of approximately 480 nursing students. In cross‐cultural adaptation of scales, it is known that participants should be selected at least five (Bryman and Cramer 2004) to 10 times (Nunnally 1978) the number of scale items. Based on the number of scale items in the study, a minimum of 105 and a maximum of 210 nursing students were tried to be reached. Nursing students at the relevant university who volunteered to participate were included in the study. The study was completed with 347 nursing students. The cultural adaptation of the scale was performed in three stages (linguistic validity, scale validity and scale reliability).

### 2.2 Instruments

The data were collected with a questionnaire form prepared by the researcher and in line with the literature (Lin and Tsai 2013; Yiin et al. 2024). The questionnaire included the ‘Descriptive Characteristics Form’ and ‘NLSE Scale’.

#### 2.2.1 Descriptive Characteristics Form

This form consists of seven items (age, gender, class, family type, feeling competent to learn nursing, economic status of the family and place of residence [school dormitory, home]) questioning the descriptive information of nursing students.

#### 2.2.2 NLSE Scale

It determines nursing students' performances and learning needs, including cognition and practice. In the original scale, there were five subdimensions, including ‘conceptual understanding (CU)’, ‘higher order cognitive skills (HC)’, ‘practical work (PW)’, ‘everyday application (EA)’ and ‘nursing communication (NC)’ and 21 questions in total. Each item was assessed on a 5‐point Likert scale (1 *strongly disagree* To 5 *strongly agree*). CU indicates nursing students' confidence in understanding subject definitions, rules and theories (e.g., I can clearly describe nursing‐related knowledge to others). HC refers to the confidence level of students in their ability to use higher order cognitive skills, such as problem‐solving and critical thinking, in the nursing approach (e.g., I can critically evaluate various methods for solving nursing problems). PW assesses students' competence in using nursing equipment, including cognitive and psychomotor abilities (e.g., I am familiar with the steps of various nursing instruments [such as blood pressure monitors and blood glucose metres]). EA addresses how confident students can apply nursing concepts and skills to situations (e.g., I can use various nursing care methods to solve problems encountered in life). NC measures students' confidence in their capacity to talk to or engage in discussions with others about nursing issues (e.g., I can discuss the nursing content I learned with my classmates). All items consisted of positive statements. A high score obtained from the scale indicates that nursing students have a high level of self‐efficacy in learning. The scale showed high internal consistency reliability with an alpha value of 0.95 (Yiin et al. 2024).

### 2.3 Linguistic Validity

Three academic translators translated the original English NLSE scale into Turkish. Afterward, these translations were brought together, common expressions were identified and the different expressions were turned into a common sentence by discussion with the translators. To ensure conceptual and linguistic equality, the Turkish version of the scale, which was created based on expert opinion, was translated back into English by a native Turkish speaker who spoke English like a native speaker and had never seen the original version of the scale before. Different academicians analysed the original and translated version of the scale, and a common opinion was reached. Thus, the translation and language validity study of the scale was completed.

### 2.4 Content Validity

Davis's (1992) method was used to determine content validity. This method grades expert opinions on four points: (a) ‘appropriate’, (b) ‘the article should be lightly revised’, (c) ‘the article should be seriously reviewed’ and (d) ‘the article is not appropriate’. In this method, the ‘Content Validity Index’ (CVI) for the item is obtained by dividing the number of experts who selected options (a) and (b) by the total number of experts, and instead of comparing this value with a statistical criterion, the value of 0.80 is accepted as the criterion. The opinions of 10 nursing educators with doctoral degrees were asked to test the content validity of the NLSE. The scale items with low points (Items 2, 6, 13 and 20) were revised in terms of content and scope and edited in line with the experts' suggestions. Each item was rated from one to four (1 = *not appropriate*; 2 = *partially appropriate but major revision is needed*; 3 = *partially appropriate but minor revision is needed*; and 4 = *very appropriate*). The CVI value is accepted to be at least 0.80 (Seçer 2018). The scale was compatible in terms of the linguistic fidelity CVI (language) = 0.95 and content fidelity CVI (content) = 0.92. As the calculated CVI value was greater than 0.80, it was determined that the content appropriateness of the NLSE scale was ensured in line with expert opinions.

### 2.5 Research Implementation

The study was conducted in two stages. A draft form was prepared in the first stage, and a pilot application was made to 20 students. The scale items were evaluated in terms of language, expression, appropriateness, typographical errors, comprehensibility and the filling time of the items. After the application, it was determined that the scale items were understandable and there were no criticisms or suggestions. The data of the students subjected to the preapplication were not included in the study. The administration announced the research to the Faculty of Health Sciences nursing students in the second stage. All nursing students at the relevant faculty who volunteered to participate were invited. The final questionnaire form was distributed face to face to 480 nursing students who met the research criteria. It took an average of 15–25 min for each participant to fill out the forms. The scale was reapplied to 50 students 2 weeks after the first data collection to evaluate the test–retest reliability of the NLSE scale.

### 2.6 Statistical Analysis

Statistical package programs (IBM SPSS Statistics for Windows [Version 25.0] and AMOS [Version 24.0]) were used for data evaluation. Kaiser–Meyer–Olkin (KMO) and Bartlett's sphericity tests were used for item analysis, and explanatory and confirmatory factor analyses were used for construct validity. In the reliability analysis, Cronbach's alpha coefficient was used to determine the internal consistency reliability. It is accepted that a time interval of 10–14 days for test and retest, a minimum sample size of 50 people and a minimum value of 0.70 are sufficient for interpreting the results (Polit and Beck 2012; Souza et al. 2017). The structural equation modelling method was also used to control the structural model. Moreover, statistical significance was set at *p* < 0.05.

### 2.7 Ethical Considerations

Permission to use the scale in the study was obtained from Yiin SJ through email. Written permissions were obtained from the local ethics committee (Decision No. 13/08, date: 17.04.2024) and the institution where the study was conducted. Informed consent was obtained from nursing students who agreed to participate in the study. All procedures involving human participants were performed according to the ethical standards of the Institutional and/or National Research Committee, the 1964 Declaration of Helsinki and its subsequent amendments.

## 3 Results

### 3.1 Characteristics of the Nursing Students

A total of 347 nursing students participated in this study. The median age of the participants was 21 years old. Among the participants, 276 (79.50%) were female, and 71 (20.50%) were male. The proportions of participants studying in the first, second, third and fourth grades were 24.80%, 21.60%, 21.30% and 32.30%, respectively. The majority of the participants had a nuclear family type (88.80%), lived in a dormitory (85.30%) and had a medium economic income level (74.10%), and about half of them perceived themselves as adequate in learning nursing (44.70%) (Table 1).

**TABLE 1:** Descriptive characteristics of the participants (*n* = 347).

	Statistics
Age, (year)	
*X ±* SD	21.10 ± 1.80
*M* (min–max)	21 (18–37)
Gender, *n* (%)	
Female	276 (%79.50)
Male	71 (%20.50)
Class, *n* (%)	
1. class	86 (%24.80)
2. class	75(%21.60)
3. class	74 (%21.30)
4. class	112 (%32.30)
Family type, *n* (%)	
Nuclear family	308 (%88.80)
Extended family	39 (%11.20)
Economic situation, *n* (%)	
Good	73 (%21.00)
Middle	257(%74.10)
Poor	17 (%4.90)
Place of residence for school education, *n* (%)	
Home	51 (%14.70)
Dormitory	296 (%85.30)
Feeling self‐inadequate in learning nursing, *n* (%)	
Inadequate	14 (%4.00)
Partially adequate	73 (%21.00)
Undecided	80 (%23.10)
Adequate	155 (%44.70)
I am quite adequate	25 (%7.20)

*Note:* Descriptive statistics were given as mean (X), standard deviation (SD), median (M), minimum (min), maximum (max), number (*n*) and percentage (%).

### 3.2 Explanatory Factor Analysis

The KMO value to test the adequacy of item distributions in the factor analysis was determined to be at a very good level (0.93). Furthermore, Barlett's test result was 3792.80 (*p* < 0.001) (Table 2).

**TABLE 2:** NLSE scale validity results.

Item No.	F1	F2	F3	F4
1	0.76			
2	0.71			
3	0.63			
5		0.75		
6		0.75		
7		0.63		
8		0.52		
18			0.69	
19			0.69	
20			0.83	
21			0.73	
9				0.71
10				0.70
11				0.73
12				0.84
13				0.82
14				0.79
15				0.58
16				0.76
17				0.57
Eıgenvalue	8.67	2.10	1.39	1.02
% of variance	41.30	51.32	57.96	62.84
Cumulative %	26.39	39.49	52.01	62.84
KMO: 0.93; *χ*2: 3792.80; df: 210				

Abbreviations: df, degree of freedom; KMO, Kaiser–Meyer–Olkin test.

### 3.3 Construct Validity

The factor loads of the questions on the scale varied between 0.52 and 0.83. According to the analysis, the first factor consisted of three items (1–3), and the factor loadings were between ‘0.63 and 0.76’. This subdimension explained 41.30% of the total variance and did not change. If an item is included in more than one dimension and the difference between the factor loading values of these dimensions is less than 0.10, in that case, these items are considered to overlap, and it is recommended that they be removed from the scale (Seçer 2018). Therefore, Item 4 in the second factor was removed from the scale. The second subfactor consisted of four items (5–8); the factor loadings were between ‘0.52 and 0.75’ and explained 51.32% of the total variance. The third factor represented the fourth subdimension of the original scale, and the scale items remained unchanged (18–21). The loadings of this factor were between ‘0.69 and 0.83’, and the factor explained 57.96% of the total variance. Unlike the original scale (Yiin et al. 2024), the third (9–14) and fourth (15–17) factors merged and formed the last subdimension in the Turkish adaptation of the scale. The loadings of this factor were between ‘0.57 and 0.84’; the factor explained 62.84% of the total variance and was renamed ‘everyday practical applications’ (Table 2).

### 3.4 Reliability Analysis

#### 3.4.1 Internal Consistency Reliability (Cronbach's Alpha)

The reliability of the Likert‐type NLSE scale was evaluated with Cronbach's α internal consistency reliability, and Cronbach's α value of the scale was determined to be 0.92. The scale score was obtained by summing the questions, and the mean was 67.91 ± 11.36 points. The Cronbach's α internal consistency reliability values of the subdimensions of the scale were 0.74, 0.78, 0.92 and 0.78 (Table 3). Considering the internal consistency reliability values obtained, it was seen that the items in the scale were sufficient to measure the perceived self‐efficacy levels of the students about learning clinical nursing. Hence, the internal consistency and reliability of the scale were ensured.

**TABLE 3:** Internal consistency reliability of the NLSE scale.

Item No.	X	SD	Scale mean if item deleted	Scale variance if item deleted	Corrected item‐total correlation	Squared multiple correlation	Cronbach's alpha if item deleted	Cronbach's alpha
1	3.41	0.87	64.49	118.88	0.50	0.40	0.92	0.74
2	3.53	0.73	64.37	119.81	0.55	0.45	0.92	
3	3.21	0.80	64.69	118.38	0.58	0.43	0.92	
5	3.68	0.74	64.23	119.95	0.53	0.46	0.92	0.78
6	3.53	0.75	64.38	119.45	0.55	0.48	0.92	
7	3.47	0.80	64.44	117.58	0.63	0.50	0.92	
8	3.57	0.90	64.33	116.78	0.59	0.45	0.92	
9	3.58	1.18	64.32	110.96	0.67	0.59	0.92	0.92
10	3.24	1.00	64.66	115.06	0.61	0.51	0.92	
11	3.07	0.92	64.83	114.42	0.70	0.61	0.92	
12	2.89	1.00	65.02	113.31	0.69	0.69	0.92	
13	3.12	0.98	64.79	113.20	0.72	0.67	0.91	
14	3.03	0.97	64.87	113.58	0.70	0.62	0.92	
15	3.48	0.83	64.43	116.18	0.68	0.52	0.92	
16	2.93	0.98	64.98	113.63	0.69	0.59	0.92	
17	3.38	0.85	64.52	116.04	0.67	0.51	0.92	
18	3.76	0.74	64.14	121.80	0.41	0.44	0.92	0.78
19	3.53	0.77	64.37	119.25	0.54	0.47	0.92	
20	3.79	0.77	64.12	120.97	0.44	0.54	0.92	
21	3.62	0.87	64.28	121.88	0.34	0.37	0.92	
Total	67.91	11.36						0.92

*Note:* Descriptive statistics were given as mean (*X*) and standard deviation (SD) values.

### 3.5 Results of Validity

#### 3.5.1 Confirmatory Factor Analysis

In confirmatory factor analysis, *χ*2/df (chi‐square to the degree of freedom), RMSEA (root mean square error of approximation), IFI (Incremental Fit Index), CFI (Comparative Fit Index), GFI (Goodness of Fit Index) and TLI (Tucker–Lewis Index) were used to evaluate the factor validity of the models. RMSEA is an index that is least affected by sample size. In this area, it is generally considered acceptable between 0.05 and 0.10. IFI, TLI, CFI and GFI fit indices of 0.90 and above are considered evidence of adequate model fit (Schermelleh‐Engel et al. 2003). The fit indices for the model obtained for the NLSE scale (*χ*2 = 411.21 df = 162) showed that the model was acceptable (Table 4).

**TABLE 4:** Statistical values for the fit of the NLSE scale model.

Scale	(χ^2^/SD)	RMSEA	IFI	CFI	GFI	TLI
Model	2.53	0.06	0.93	0.93	0.88	0.91

Abbreviations: CFI, Comparative Fit Index; GFI, Goodness of Fit Index; IFI, Incremental Fit Index; RMSEA, root mean square error of approximation; TLI, Tucket–Lewis Index.

**FIGURE 1:**
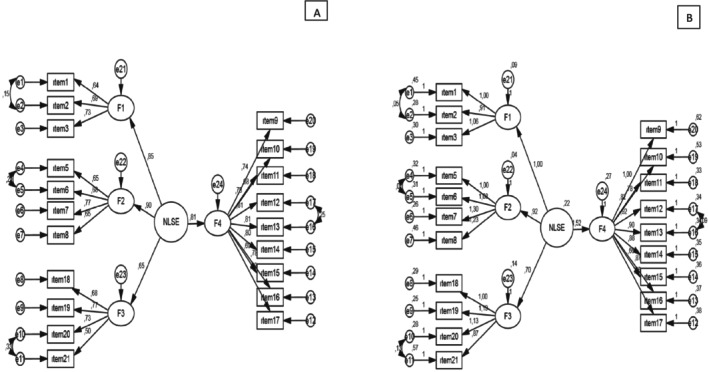
Confirmatory factor analysis model with standardized (A) and unstandardised (B) coefficients of NLSE scale.

The 20‐item NLSE scale was subjected to a CFA. The model is presented in Figure 1. Each of the path coefficients calculated for the 20 questions of the scale was statistically significant (*p* < 0.05) (Figure 1).

### 3.6 Floor and Ceiling Effects

The floor and ceiling effects of the NLSE scale were analysed. The floor effect is the proportion of people scoring 1 point for all items, totalling 21 points, whereas the ceiling effect is the proportion of people scoring 5 points, totalling 105 points, and it is undesirable for this proportion to be above 15% (Pontes and Griffiths 2015). When the distribution of the scores obtained was analysed, it was seen that no one scored 21 or 45 points. Moreover, item discrimination analysis was performed for the total score and subscales of the NLSE. For 37 items, the item analysis technique based on the difference between the upper and lower group means (based on the internal consistency criterion) was applied. With this method, item NLSE total score and subscale scores were ranked from highest to lowest. According to this ranking, 94 people constituting the first 27% of the group of 347 people were determined as the upper group, and 94 people constituting the last 27% were determined as the lower group. The difference between the means of the 27% lower and upper groups for each scale item was examined with the *t* test for independent groups. As a result of the item discrimination analysis (lower–upper 27% group comparison), it was determined that the significant difference obtained provided the item discrimination power of the NLSE total score and its subdimensions (*p <* 0.05) (Table 5).

**TABLE 5:** Item discrimination analysis of NLSE total score and subdimensions (*n* = 94).

	Floor		Ceiling		*t*	*p*
	X	SD	X	SD		
Conceptual understanding	8.37	1.76	11.88	1.18	**−16.02**	**0.00**
Higher order cognitive skills	11.79	2.68	16.18	1.58	**−13.65**	**0.00**
Nursing communication	19.94	4.95	35.50	2.89	**−26.29**	**0.00**
Everyday practical applications	12.89	2.89	16.57	1.68	**−10.65**	**0.00**
Scale total	52.99	7.49	80.14	4.64	**−29.84**	**0.00**

*Note:* Descriptive statistics are given as mean (X) and standard deviation (SD) values. The bolded sections are statistically significant (*p* < 0.05).

Abbreviation: *t*, Student's *t* test.

### 3.7 Retest Analysis

It was determined that the initial and final total test statistics were significant (*p* = 0.01) and the correlation was excellent (*r* = 0.86, *p* < 0.001). Furthermore, the intraclass correlation coefficient (ICC) value was 0.92 (0.87–0.95), which was significant (Table 6).

**TABLE 6:** NLSE scale retest analysis results (*n* = 50).

	Pretest	Retest	z	Test (*p*)	Total correlation	ICC (%95 GA)
Conceptual understanding	10.62 ± 1.45	10.72 ± 1.61	0.81	0.41	**0.74**	**0.85 (0.76–0.91)**
	11 (7–15)	11 (7–15)				
Higher order cognitive skills	14.69 ± 1.36	14.69 ± 1.36	−1.73	0.08	**0.98**	**0.99 (0.99–0.99)**
	15 (11–17)	15 (11–17)				
Nursing communication	31.46 ± 3.83	32.25 ± 4.09	2.41	**0.01**	**0.79**	**0.88 (0.80–0.92)**
	32 (21–39)	33 (24–40)				
Everyday practical applications	15.24 ± 2.20	15.42 ± 2.52	0.89	0.36	**0.75**	**0.85 (0.75–0.91)**
	16 (6–20)	16 (5–20)				
Scale total	72.03 ± 7.01	73.10 ± 7.93	2.48	**0.01**	**0.86**	**0.92 (0.87–0.95)**
	73 (56–84)	74 (55–89)				

*Note:* Summary statistics are given as mean ± standard deviation. Sections in bold are statistically significant (*p* < 0.05).

Abbreviations: ICC, intraclass correlation coefficient; *z*, Wilcoxon test.

## 4 Discussion

Perceived self‐efficacy helps self‐regulate unsuccessful and stubborn behaviours (Bandura 1982). Bandura (1997) theorized that individuals with higher levels of education will learn more through formal education and, thus, have more opportunities to learn to cope with problems. This study tested the applicability of the NLSE scale, which measures student nurse's self‐efficacy in learning, in the Turkish population. The NLSE scale developed to determine nursing students' self‐efficacy in learning was appropriate for nursing students in the Turkish population. Adapting the NLSE scale to the Turkish culture showed good internal consistency and reliability and provided valid and reliable evidence with an acceptable level of agreement.

Some modifications were identified while testing the NLSE scale in the Turkish population. Any item that appears in more than one factor with a difference of less than 0.10 is considered an overlapping item, and these items are removed from the scale (Büyüköztürk 2002). In the EFA, the factor loading of the fourth item was 0.49 in the first subfactor and 0.48 in the second subfactor. Because the difference between the factors of the fourth item was less than 0.10, this item was removed from the scale. We believe that Turkish nursing students were confused about this item. In the original scale (Yiin et al. 2024), there were five items, including the fourth item in the second factor, whereas there were four items in the second subfactor in the Turkish adaptation of the scale after removing this item.

In nursing education programmes, theoretical and clinical learning environments play an important role in acquiring professional skills (Panda et al. 2021). Unlike the original scale, it was determined that the ‘practical work’ and ‘everyday practice’ factors merged in the Turkish adaptation analyses. Therefore, these combined factors were renamed ‘everyday practical practices’ to represent both factors. Nursing students associate facts, concepts and events to decide on the use of nursing equipment. Students who are confident in their nursing concepts and skills are likely to feel competent in using equipment (Cura et al. 2020). It has been stated that the availability of clinical equipment and materials affects the quality of nursing skills performances (Relloso et al. 2021). This factor, renamed ‘everyday practical practices’, consisted of nine items. The ‘understanding the concept’ and ‘nursing communication’ factors of the scale were kept as they were in the original version.

The total variance explained by the 21 items in the original scale was 77.72% (Yiin et al. 2024), the total variance explained by the 20 items in the current study was 62.84% and the variance explained by the different self‐efficacy scales was 79.91% (Yavuzalp and Bahcivan 2020), and these values were quite good. Factor loadings also revealed a strong construct validity for the final version of the four‐factor model. The *χ*2/SD < 3.0, RMSEA < 0.08 and GFI CFI, IFI and TLI values > 0.90 (Gefen et al. 2000) indicated that the EFA model of the scale applied to the Turkish population was acceptable. Model E in the original scale and structural equation modelling for the Turkish population showed a close fit.

The Cronbach's α value of the original scale (0.95) (Yiin et al. 2024), the Cronbach's α value of the present study (0.92) and the Cronbach's α value evaluating self‐efficacy in a different field (0.88) (Wang et al. 2018) were quite close to each other. These tests prove that the NLSE scale is a valid and acceptable measurement tool for the Turkish population.

A retest analysis was conducted to test construct validity and invariance over time further. The values in the original version of the scale (Yiin et al. 2024) and in research evaluating the self‐efficacy of Taiwanese and American students (Wang et al. 2018) showed a good correlation, similar to the present study. These results show that the NLSE scale is adaptable and applicable to the Turkish population, and its construct validity was once again supported.

### 4.1 Conclusion and Recommendations

The results of this study showed that the NLSE scale is a valid and reliable tool for assessing Turkish nursing students' self‐efficacy in learning. Evaluating nursing students' perceived self‐efficacy related to nursing professional skills using a standardized measurement tool allows educators to provide quality and successful skill acquisition. At the same time, the NLSE scale, whose validity and reliability were tested, is useful for discovering the determinants affecting self‐efficacy and measuring the adequacy of educational interventions. Assessment of self‐efficacy in learning the nursing profession will help test educational interventions' effectiveness. There is a need to test the psychometric properties of this scale for cultural adaptation in other countries.

### 4.2 Strengths and Limitations of the Study

The data in this study were obtained from the responses of nursing students studying only in one region of Türkiye. Therefore, further validity tests and scale improvement studies based on cultural or systematic differences regarding self‐efficacy in learning nursing are needed. Another limitation of the study is that the results were valid only for the nursing students who participated in the questionnaire, and it was not possible to generalize the results to nursing students in all provinces of Türkiye.

### 4.3 Implications for Nursing Practice

Nursing students should have high self‐efficacy to learn the nursing profession successfully. Self‐efficacy in learning nursing should be evaluated with a valid and reliable measurement tool. In previous studies, the self‐efficacy levels of individuals have been investigated in different fields, and the importance of examining self‐efficacy has been revealed. This study provides an opportunity to evaluate the self‐efficacy of Turkish nursing students in learning the profession. This study provides comprehensive, applicable and acceptable information for assessing Turkish nursing students' self‐efficacy levels in learning the profession. Moreover, this study can serve as a basis for further studies with a larger sample in Türkiye and other countries.

## Author Contributions

**Özlem Şahin Akboğa:** concept, design, supervision, resources, materials, data collection and/or processing, analysis and/or interpretation, literature search, writing manuscript, critical review.

## Disclosure

We the undersigned declare that this manuscript is original, has not been published before and is not currently being considered for publication elsewhere. We confirm that the manuscript has been read and approved by all named authors and that there are no other persons who satisfied the criteria for authorship but are not listed. We further confirm that the order of authors listed in the manuscript has been approved by all of us. We understand that the corresponding author is the sole contact for the editorial process. She is responsible for communicating with the other authors about progress, submissions of revisions and final approval of proofs.

## Conflicts of Interest

The author declares no conflicts of interest.

## Data Availability

The data that support the findings of this study are available from the corresponding author upon reasonable request.
